# Importance of an Early HIV Antibody Differentiation Immunoassay for Detection of Dual Infection with HIV-1 and HIV-2

**DOI:** 10.1371/journal.pone.0157690

**Published:** 2016-06-16

**Authors:** Andrea Zbinden, Roland Dürig, Cyril Shah, Jürg Böni, Jörg Schüpbach

**Affiliations:** 1 Institute of Medical Virology, University of Zurich, CH-8057, Zurich, Switzerland; 2 Maihof Praxis, CH-6004, Lucerne, Switzerland; 3 Swiss National Center for Retroviruses, University of Zurich, CH-8057, Zurich, Switzerland; Waseda University, JAPAN

## Abstract

**Background:**

HIV-2 is primarily endemic in West Africa and India, however, in time of global migration, a possible HIV-2 infection or co-infection with HIV-1 should be recognized right at the time of HIV diagnosis, in order to enable optimized antiretroviral treatment. Laboratory HIV testing consists of a combined HIV1/2/O antibody + antigen screening test and subsequent confirmation and type differentiation by a serological test formatted as a multi-line or multi-spot assay. CDC has proposed a revised alternative HIV diagnostic strategy which, in case of a reactive result in a combined HIV1/2/O antibody + antigen screening test, comprises an HIV-1 nucleic acid test (NAT) for HIV confirmation instead of an antibody differentiation immunoassay (ADI). Only a negative NAT must be further investigated by an ADI, thus saving expenses for ADI in most instances. We have investigated this alternative strategy with respect to its recognition of dual HIV-1 and HIV-2 infection.

**Methods and Results:**

Anonymized data of HIV notifications of patients newly diagnosed with HIV in Switzerland between 2007 and 2014 were analysed retrospectively. In a total of 4'679 notifications, we found 35 HIV-2 infections, 9 (25.7%) of which were dually infected with HIV-1. In 7 of the 9 dual HIV-1 and HIV-2 infections, HIV-1 RNA testing at the time of HIV diagnosis was positive with concentrations from 102 to 94'300 copies/mL plasma. HIV-1 RNA data were not available for the other two cases.

**Conclusions:**

The alternative CDC strategy would have missed the concomitant HIV-2 infection in at least 7, but probably even more, of the 9 dually infected patients, as the detectable HIV-1 RNA would have precluded a supplemental ADI. Early ADI is mandatory for diagnosis of dual HIV-1/HIV-2 infection and guidance of appropriate therapy.

## Introduction

Accurate laboratory diagnosis of HIV is essential for patient management and treatment. The current HIV diagnostic algorithm presented by the U.S. Centers for Disease Control and Prevention (CDC) in June 2014 consists of a combined screening immunoassay, which detects both p24 antigen and HIV antibodies, and subsequent confirmation and type differentiation by a serological test formatted as a multi-line or multi-spot assay [1]. If this HIV-1/HIV-2 antibody differentiation immunoassay (ADI) is nonreactive or indeterminate, an HIV-1 nucleic acid test (NAT) is recommended [1]. An alternative testing algorithm using HIV-1 NAT as the confirmatory test after a reactive screening assay instead of an ADI was also proposed [1]. In this strategy, only a negative HIV-1 NAT result would be followed by an ADI, thus saving expenses for ADI in most instances. This alternative testing algorithm was evaluated previously and identified a higher proportion of HIV infections than did ADI-based confirmation; however, it does not allow for differentiation of acute from established HIV infection [2]. A further confirmatory test was recommended to resolve discrepancies and avoid incorrect diagnoses [1, 2].

Based on a well-documented case of dual HIV-1 and HIV-2 infection where HIV-2 would have been missed when following the alternative HIV testing algorithm we investigated how many such dual infections would have been missed in recent years.

## Material and Methods

### Data origin and ethics statement

In Switzerland, anonymized HIV notifications for patients newly diagnosed with HIV infection were reported to the Swiss Federal Office of Public Health (SFOPH) since 2007. There were two types of notifications for each patient: (i) a laboratory notification with the diagnostic test data sent in by one of the 11 regional HIV notification labs in Switzerland or the Swiss National Center for Retroviruses of Switzerland, and (ii) a supplemental clinical notification forwarded by the patient’s attending physician, with epidemiologic and clinical information relevant in the context of HIV infection [3]. No informed consent was required, since both types of anonymous notifications are imposed by Swiss federal law. Notifications, which include the results of all HIV laboratory tests performed at the time of HIV diagnosis were available since September 2007 [3]. We used these data for a search for dually infected patients. All data in this study were contained in anonymized, legally mandatory HIV notifications to the SFOPH.

### Laboratory testing methods

For confirmation of HIV infection and differentiation between HIV-1 and HIV-2, the line immunoassay, INNO-LIA HIV I/II Score (Fujirebio, Ghent, Belgium) (Inno-Lia), had been used in all patients at time of diagnosis. HIV-1 viral RNA plasma load had been measured by the Cobas AmpliPrep/-Taqman HIV-1 version 2.0 (Roche Diagnostics, Rotkreuz, Switzerland) with a detection limit of 20 copies/mL. HIV-2 RNA plasma load was assessed by a specific HIV-2 RT-PCR with a detection limit of 100 copies/mL [4]. An ultra-sensitive high input assay (Mega) PCR for verification of dual HIV-1/HIV-2 infection was performed with DNA extracted from peripheral blood mononuclear cells (PBMC) as described [5].

## Results

### Case presentation of a HIV-1 and HIV-2 dually infected patient

**Fig 1:**
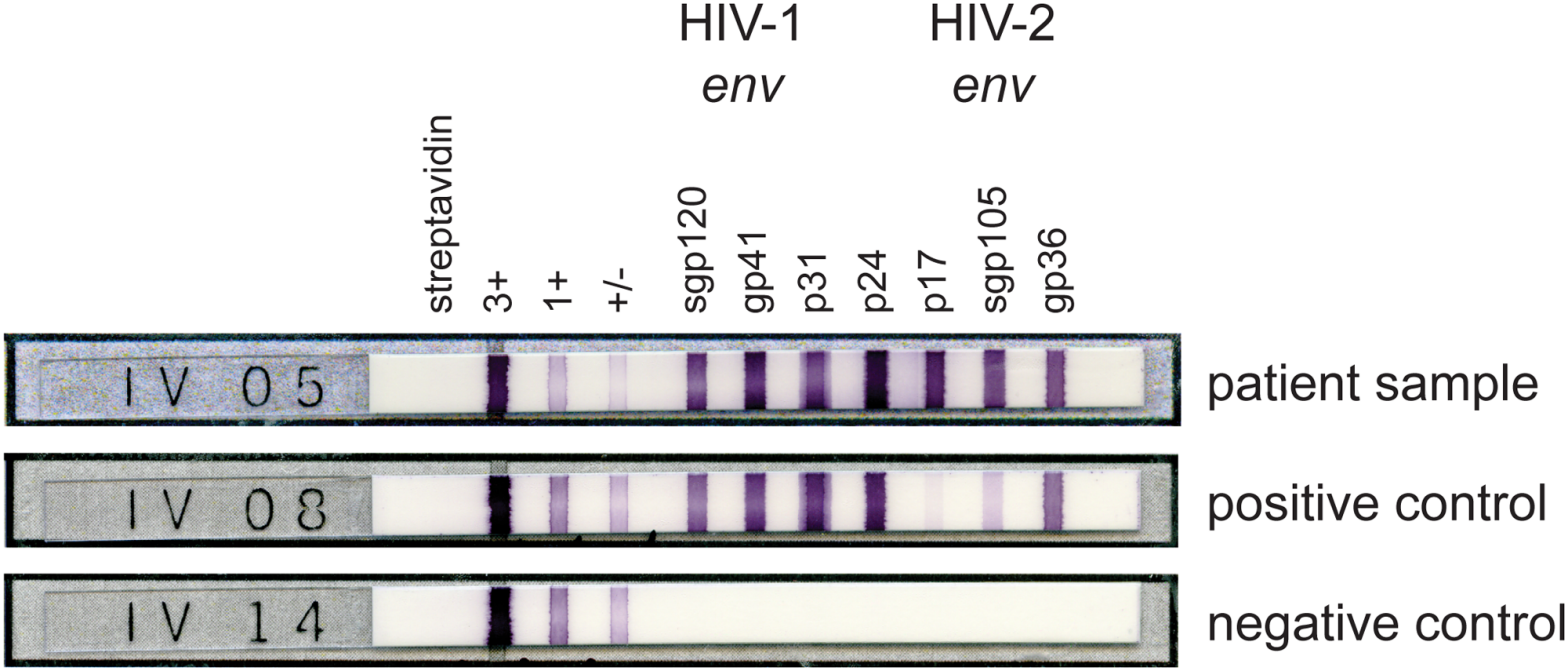
Line immunoassay of patient 1 showing seropositivity for both HIV-1 and HIV-2. Patient sample: specific HIV-1 envelope antibodies (sgp120 and gp41) and specific HIV-2 envelope antibodies (gp36 and sgp105) are present. Positive and negative strip controls are shown.

In April 2012, a 27-year-old man (patient 1) originating from Guinea Bissau presented to a hospital in Switzerland with fever and multiple lymphadenopathy. A fourth-generation HIV-1/2 antibody/antigen combination EIA screening test was reactive. Testing for p24 antigen alone was negative. For confirmation of HIV infection and differentiation between HIV-1 and HIV-2, the Inno-Lia immunoassay revealed a band pattern indicating HIV-1 and HIV-2 co-infection (Fig 1). The plasma HIV-1 RNA load was high with 88’000 copies/mL. The baseline CD4+ T cell count was 48 cells/μL (CD4 percentage, 5.5%). The HIV-2 RNA plasma load was negative. Mega-PCR detected HIV-2 proviral DNA in PBMC of a second sample, thus confirming a dual infection with HIV-1 and HIV-2. Genetic drug resistance testing of HIV-1 revealed HIV-1 of the recombinant clade CRF02_AG, and showed no mutations in the reverse transcriptase region of the *pol* gene and only one mutation in the protease region, 20I, potentially conferring low-level resistance to protease inhibitors (PI). After two weeks on antiretroviral treatment, the HIV-1 RNA in plasma had decreased to 5'251 copies/mL. The patient then returned to his original country, and no follow-up samples were available.

### Frequency of HIV-1 and HIV-2 dual infections in Switzerland

Among 4'679 notifications received between Sept 2007 and Dec 2014, 4'644 (99.3%) patients were infected with HIV-1 and 35 patients (0.7%) were infected with HIV-2, respectively. Including patient 1, a dual infection with HIV-1 and HIV-2 was suspected in 9 out of 35 patients (25.7%), based on serological data as shown by Inno-Lia (Table 1). Seven of these 9 patients had a detectable or high concentration of HIV-1 RNA in plasma, while the remaining two had not been tested. Where tested, HIV-2 RNA was undetectable or only weakly positive (Table 1).

**Table 1:** Diagnostic results of patients with dual HIV-1/HIV-2 infection (n = 9).

Patient	HIV line immunoassay (reactivity)	HIV-1 RNA [copies/ml]	HIV-2 RNA	HIV-2 DNA high input
1	HIV-2: sgp36 3+, sgp105 3+ HIV-1: sgp120 3+, gp41 3+, p31 3+, p24 3+, p17 3+	88’000	undetected	positive
2	HIV-2: sgp36 3+, sgp105 2+ HIV-1: sgp120 3+, gp41 3+, p31 2+, p24 4+, p17 4+	94’300	undetected	nd^a^
3	HIV-2: sgp36 4+, sgp105 4+ HIV-1: sgp120 2+, gp41 3+, p31 3+, p24 3+, p17 3+	3'690	weak-positive	nd
4	HIV-2: sgp36 3+, sgp105 3+ HIV-1: sgp120 3+, gp41 3+, p31 3+, p24 3+, p17 3+	21’000	undetected	nd
5	HIV-2: sgp36 3+, sgp105 3+ HIV-1: sgp120 3+, gp41 3+, p31 3+, p24 3+, p17 3+	102	undetected	positive
6	HIV-2: sgp36 3+, sgp105 4+ HIV-1: sgp120 2+, gp41 3+, p31 2+, p24 4+, p17 3+	nd	nd	nd
7	HIV-2: sgp36 2+, sgp105 2+ HIV-1: sgp120 3+, gp41 3+, p31 3+, p24 3+, p17 2+	4039	nd	nd
8	HIV-2: sgp36 3+, sgp105 3+ HIV-1: sgp120 2+, gp41 2+, p31 2+, p24 3+, p17 0	nd	nd	nd
9	HIV-2: sgp36 1+, sgp105 1+ HIV-1: sgp120 2+, gp41 3+, p31 2+, p24 3+, p17 2+	4618	nd	nd

^a^nd, not done.

## Discussion

HIV-2 is most prevalent in West Africa and India, however, there are reports of HIV-2 in Europe and the United States due to migration [6–8]. Switzerland or Austria are low-endemic countries; notably, some of the HIV-2 infections are dual infections with HIV-1 [3, 9, 10]. Our retrospective analysis of all HIV notifications for patients newly diagnosed with HIV infection from Sept 2007 to Dec 2014 shows that a quarter of all patients with HIV-2 infection were additionally infected with HIV-1. Moreover, most of the HIV-1 plus HIV-2 infections displayed rather high HIV-1 viral loads right at the time of HIV diagnosis (ranging from 102 to 94'300 copies/mL plasma). By applying the alternative HIV diagnostic algorithm presented from CDC [1], which requires an ADI only in case of undetectable HIV-1 RNA in plasma, at least 7 (20%) out of 35 HIV-2 infections would have been missed.

Previous observations have suggested that HIV-2 may exert a protective effect against HIV-1 and it was thought that there is no simultaneous virus replication in dually infected patients [11]. A review of the literature shows, however, that the combination of a high HIV-1 plasma load with a low or undetectable HIV-2 plasma load, as we have observed in most of our dual infections, is not rare [12]. Andersson et al. showed a significantly lower viral load set-point for HIV-2 than for HIV-1 in a number of patients [13]. Moreover, with regard to the alleged protective effect of HIV-2, a higher risk for acquiring HIV-1 was observed for HIV-2 infected individuals [14]. Schutten et al. described two patients with dual HIV-1 and HIV-2 infections, who displayed active replication of both viruses and had a clinical failure after ART against HIV-1 [15].

Early identification of HIV-2 is critical for patient management as all viruses of this type are naturally resistant to the entire class of non-nucleoside reverse transcriptase inhibitors. Moreover, mutations conferring reduced susceptibility of HIV-2 to PIs were detected in dually infected patients, leading to HIV-2 viral plasma rebounds or persisting plasma viral loads [16, 17].

Verification of a serologically suspected HIV-2 infection still is challenging since current commercial methods do not allow for detection of HIV-2 RNA loads in plasma samples, and proper quantification of HIV-2 in plasma is complicated by limited HIV-2 standards and the genetic diversity of specific HIV-2 strains [4]. Moreover, as many HIV-2 infected patients are asymptomatic and have an undetectable or significantly lower HIV-2 RNA load in plasma than HIV-1 RNA [12, 13], the diagnostic sensitivity of NAT for HIV-2 RNA is low and a negative test result does not exclude HIV-2 infection. Given the low sensitivity of standard PCR for detection of proviral HIV-2 DNA, a Mega-PCR was developed and successfully applied in the past [5]. However, in most cases presented here, Mega-PCR was not performed and the diagnosis of HIV-2 was based on Inno-Lia alone.

We conclude that a significant number of HIV-1 and HIV-2 dual infections would be underreported when following the alternative strategy of HIV diagnosis as proposed by CDC. Application of an ADI early at HIV diagnosis is highly recommended to identify HIV-2 co-infections and guidance of appropriate therapy irrespective of epidemic data.
